# Cystic carcinoid tumor of the pancreas diagnosed by endoscopic ultrasound-guided fine needle aspiration of the cystic wall: an unusual presentation and diagnosis

**DOI:** 10.1590/S1679-45082014AI2516

**Published:** 2014

**Authors:** Rogério Colaiacovo, Ana Carolina Figueiredo de Castro, Ricardo Leite Ganc, Christina Shiang, Renée Zon Filippi, Ângelo Paulo Ferrari

**Affiliations:** 1 Department of Endoscopy, Hospital Israelita Albert Einstein, São Paulo, SP, Brazil

Cystic carcinoid tumors of the pancreas represent a subgroup of malignant potential and difficult diagnosis.^(1,2)^

Endoscopic ultrasound-guided fine needle aspiration (EUS-FNA) is an effective tool to evaluate these lesions.^(1–4)^

A 52-year old man was referred to the *Hospital Israelita Albert Einstein* to investigate a pancreatic cyst. Endoscopic ultrasound revealed a cystic lesion, measuring 2cm in the pancreatic tail, without septations and communication with the pancreatic duct (Figures 1 and 2). The FNA fluid showed normal amylase (67U/L) and low CEA (11,2ng/mL) levels.

**Figure 1:**
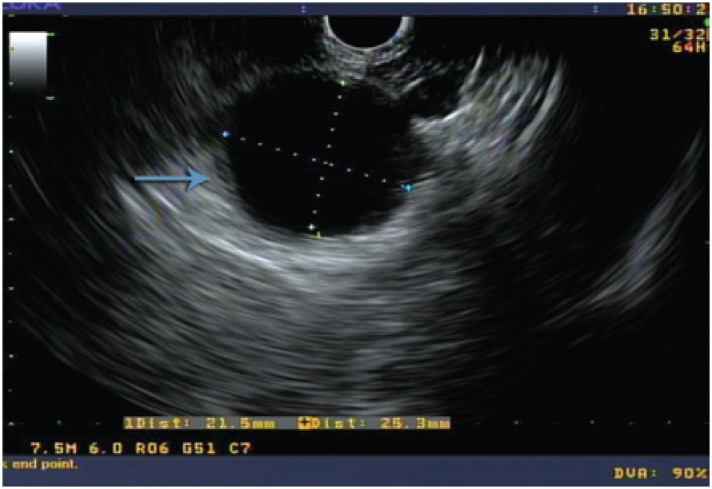
Neuroendocrine cyst

**Figure 2:**
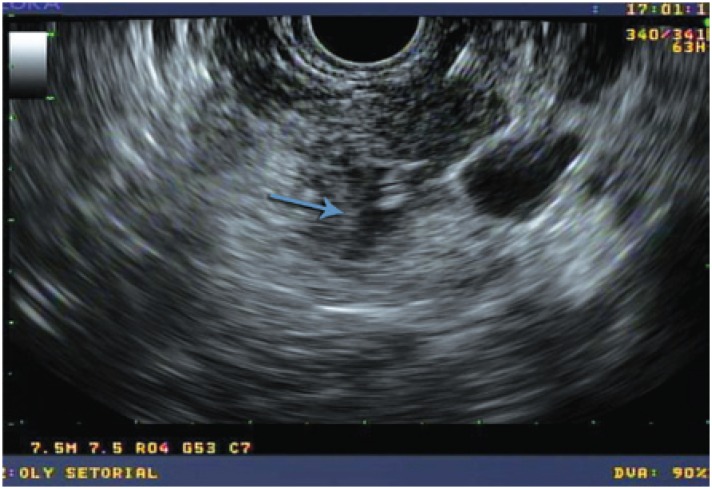
After fine needle aspiration

Histological examination of fragments of the cystic wall obtained by FNA (Figure 3) revealed a carcinoid tumor, confirmed by chromogranin and synaptofisin immunohistochemistry analyses (Figure 4).

**Figure 3:**
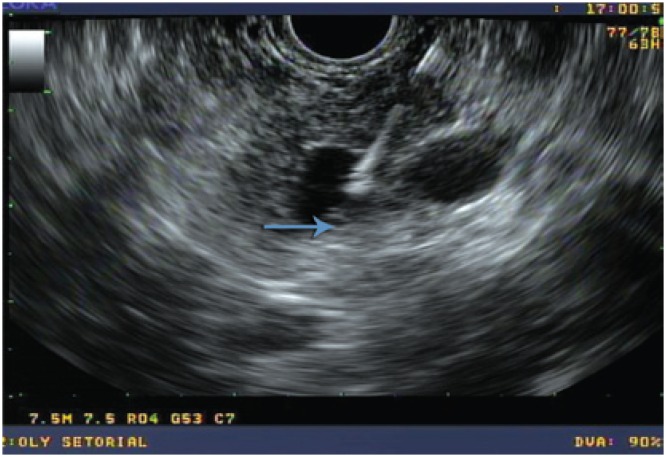
Endoscopic ultrasound-guided fine needle aspiration of the cyst wall

**Figure 4:**
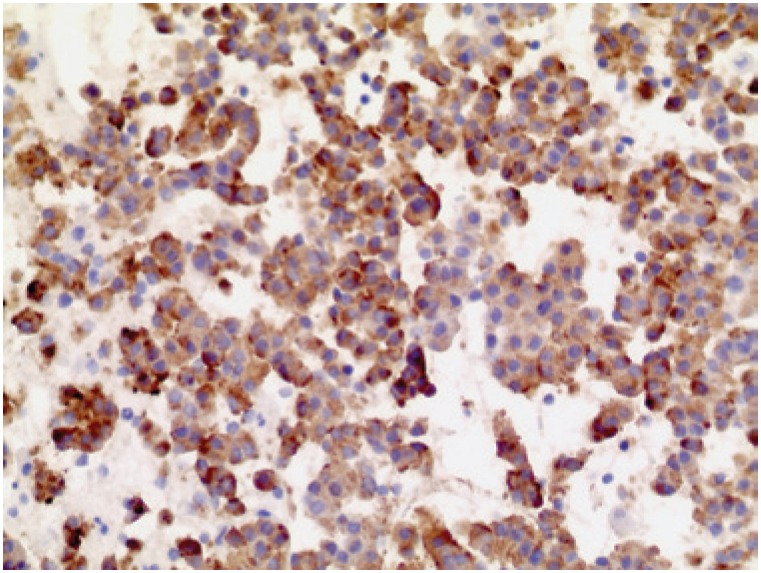
Cell-block of the pancreas shows numerous epithelial cells with rounded nucleus, “salt and pepper” chromatin and eosinophilic cytoplasm (Hematoxylin and eosin stain, 10x). Immunohistochemistry positive for neuroendocrine tumor

## DISCUSSION

The preoperative diagnosis of cystic pancreatic carcinoid tumor is important due to their malignant potential and possibility of ressection.^(5)^

Computed tomography (CT) and EUS may not help making diagnosis, since the radiological aspect is often interpreted as a pancreatic mucinous cystadenoma, such as in this report.

EUS-FNA is a highly accurate method to diagnose pancreatic carcinoid tumors.^(2–4)^ The few studies available show a high agreement between cytology and pathology.^(2–4)^

In this report a rare lesion is described, and the diagnosis was possible only after the histological study of the cystic wall fragments obtained by EUS-FNA.

This case report shows the efficacy of EUS-FNA in an unusual diagnosis.
